# Supplementary material for: Development and validation of a scoring system to predict mortality in patients hospitalized with COVID-19: A retrospective cohort study in two large hospitals in Ecuador
Source: PLoS One. 2023 Jul 17;18(7):e0288106. doi: 10.1371/journal.pone.0288106 (PMC10351692; doi:10.1371/journal.pone.0288106)
Supplement: S2 Table — (DOCX) [file pone.0288106.s003.docx]

*S2 Table. Comparison of characteristics between imputed and non-imputed data sets (derivation cohort).*

| **Variable** | **Non imputed data set**  **n=2497** | **After 10 imputations**  **n=2497** |
| --- | --- | --- |
| Male sex, *n(%)* | 1569 (63) | 1569 (63) |
| Age, *mean (SD)* | 56.5 (16.3) | 56.5 (16.6) |
| Anthropometry |  |  |
| *Weight in Kg, mean (SD)* | 71.6 (15.1) | 68.9 (15.4) |
| *Height in m, mean (SD)* | 1.62 (0.09) | 1.60 (0.11) |
| *BMI in Kg/m^2^, mean (SD)* | 27.5 (3.9) | 27.0 (6.0) |
| Vital signs |  |  |
| *Respiratory rate, mean (SD)* | 21.8 (3.9) | 22.4 (4.6) |
| *Cardiac rate, mean (SD)* | 84.9 (14.8) | 85.0 (15.0) |
| *SBP in mmHg, mean (SD)* | 118.4 (16.4) | 119.5 (17.5) |
| *DBP in mmHg, mean (SD)* | 70.8 (10.4) | 70.4 (11.1) |
| *Oxygen saturation, mean (SD)* | 97.3 (3.4) | 90.9 (3.9) |
| *Body temperature in Celsius degrees, mean (SD)* | 36.8 (0.6) | 36.8 (0.6) |
| Laboratory parameters |  |  |
| *Glucose in mg/dL, mean (SD)* | 132.2 (75.0) | 132.4 (74.9) |
| *Creatinine in mg/dL, mean (SD)* | 1.2 (1.6) | 1.2 (1.7) |
| *BUN in mg/dL, mean (SD)* | 18.6 (16.0) | 18.5 (16.1) |
| *AST in U/L, mean (SD)* | 49.0 (60.9) | 48.7 (63.8) |
| *ALT in U/L, mean (SD)* | 52.5 (58.3) | 52.1 (60.2) |
| *LDH en U/L, mean (SD)* | 295.4 (306.2) | 272.4 (256.0) |
| *CPK in U/L, mean (SD)* | 180.3 (456.7) | 180.2 (456.7) |
| *Ferritin in ng/ml, mean (SD)* | 634.1 (2071.5) | 574.1 (1115.3) |
| *C-reactive protein in mg/L, mean (SD)* | 51.7 (68.9) | 49.2 (66.7) |
| *Procalcitonin in ng/mL, mean (SD)* | 4.6 (61.9) | 2.59 (41.8) |
| *Arterial pH, mean (SD)* | 7.39 (0.09) | 7.38 (0.09) |
| *pCO_2_ in mmHg, mean (SD)* | 30.5 (8.2) | 30.8 (9.6) |
| *White blood cell count x10^3^/μL, mean (SD)* | 9.16 (5.25) | 9.27 (5.19) |
| *Lymphocytes cell count x10^3^/μL, media (DE)* | 1.20 (0.60) | 1.20 (0.63) |
| *Red blood cells count x10^6^/μL , mean (SD)* | 4.87 (0.81) | 4.87 (0.82) |
| *Hematocrit in percentage, mean (SD)* | 42.5 (6.4) | 42.5 (6.5) |
| *Hemoglobin levels in g/dL, mean (SD)* | 14.6 (2.3) | 14.5 (2.4) |
| *Platelet count x10^3^/μL, mean (SD)* | 274 (127) | 275 (119) |
| *PT in seconds, mean (SD)* | 13.8 (4.4) | 13.5 (3.2) |
| *PTT in seconds, mean (SD)* | 33.5 (12.3) | 34 (12) |
| *D-dimer in ng/mL, mean (SD)* | 28.8 (211.5) | 49.0 (221.5) |
| *SIRS, n (%)* | 2218 (88.8) | 2218 (88.8) |
| *1 point, n (%)* | 279 (19) | 324 (13) |
| *2 points, n (%)* | 766 (52) | 1423 (57) |
| *3 points, n (%)* | 408 (27) | 699 (28) |
| *4 points, n (%)* | 35 (2) | 51 (2) |
| *Clinical conditions at admission* |  |  |
| *High blood pressure (SBP≥140 mmHg or DBP≥90 mmHg), n (%)* | 181 (11.8) | 340 (13.6) |
| *Low blood pressure (SBP <90 mmHg), n (%)* | 23 (2) | 52 (2.1) |
| *Upper than normal creatinine levels (creatinine>1.35 mg/dL if men or Creatinine>1.04 if women)* | 382 (15) | 409 (16) |
| *Anemia, n (%)****^a^*** | 498 (20) | 511 (20) |
| *Obesity (≥30Kg/m^2^), n (%)* | 19 (26) | 906 (46) |
| BMI= body mass index, AST=Alanine transaminase, ALT= Aspartate transaminase, LDH= Lactic Acid Dehydrogenase, BUN=blood urea nitrogen, CPK= Creatinine phosphokinase, PT=prothrombin time, PTT=partial thromboplastin time, SIRS=systemic inflammatory response syndrome and it means that there is, at least, two of the next criteria: Body temperature over 38 or under 36 degrees Celsius, heart rate greater than 90 beats/minute, respiratory rate greater than 20 breaths/minute or partial pressure of CO_2_ less than 32 mmHg, leucocyte count >12 x 10^3^ or less than 4 x 10^3^ /μL or over 10% immature forms or bands.  ***^a^***= When red blood cells low count was <3.9 for females, and when it was <4.4 for males in Guayaquil; or, when it was <4.3 for females, and when it was <5.0 for males in Quito | | |
