# Supplementary material for: Development and validation of a scoring system to predict mortality in patients hospitalized with COVID-19: A retrospective cohort study in two large hospitals in Ecuador
Source: PLoS One. 2023 Jul 17;18(7):e0288106. doi: 10.1371/journal.pone.0288106 (PMC10351692; doi:10.1371/journal.pone.0288106)
Supplement: S3 Table — (DOCX) [file pone.0288106.s004.docx]

*S3 Table. Comparison of the discriminatory power of the score between hospitals and between sex categories, calculating the Area under the ROC curve in the validation cohort.*

|  | **n** | **AUC** | **[95% Conf. Interval]** | |
| --- | --- | --- | --- | --- |
| Total sample (validation cohort) | 2565 | 0.876 | 0.822 | 0.930 |
| Hospitals *(altitude)* |  |  |  |  |
| *Guayaquil Hospital (0 m)* | 978 | 0.742 | 0.712 | 0.773 |
| *Quito Hospital (2885 m)* | 1587 | 0.848 | 0.823 | 0.874 |
| Sex categories |  |  |  |  |
| *Male* | 1594 | 0.877 | 0.819 | 0.935 |
| *Female* | 971 | 0.829 | 0.671 | 0.986 |
