# Supplementary material for: Development and validation of a scoring system to predict mortality in patients hospitalized with COVID-19: A retrospective cohort study in two large hospitals in Ecuador
Source: PLoS One. 2023 Jul 17;18(7):e0288106. doi: 10.1371/journal.pone.0288106 (PMC10351692; doi:10.1371/journal.pone.0288106)
Supplement: S4 Table — (DOCX) [file pone.0288106.s005.docx]

**S4 Table. - Summary of the Cox Model Calibration Results for Quito**

| **Test** | **Variable** | **Coefficient** | **Standard Error** | **Z-score** | **P-value** | **95% Confidence Interval** |
| --- | --- | --- | --- | --- | --- | --- |
| Test 1 | _times (1) | -0.0891 | 0.5176 | -0.17 | 0.863 | -1.1035, 0.9254 |
| Test 1 | _times (2) | -0.0832 | 0.3581 | -0.23 | 0.816 | -0.7851, 0.6187 |
| Test 1 | Chi-square test |  |  |  | 0.9712 |  |
| Test 2 | _clogF | 0.9630 | 0.2842 | 3.39 | 0.001 | 0.4060, 1.5199 |
| Test 2 | _times (1) | -0.1389 | 0.7666 | -0.18 | 0.856 | -1.6413, 1.3635 |
| Test 2 | _times (2) | -0.1229 | 0.5541 | -0.22 | 0.824 | -1.2089, 0.9630 |
| Test 2 | Chi-square test |  |  |  | 0.8963 |  |
| Test 3 | Chi-square test |  |  |  | 0.9965 |  |
| Test 4 | 2._times | 0.1727 | 0.9667 | 0.18 | 0.858 | -1.7220, |
| Table notes:   1. Coefficients, standard errors, Z-scores, p-values, and 95% confidence intervals were obtained using the stcoxcal command in Stata. 2. All p-values are greater than 0.05, indicating a good calibration of the model for the city of Quito. 3. The null hypothesis of the Chi-square tests could not be rejected in all cases, further suggesting a well-calibrated model. 4. The variables starting with '_times' represent different time points, 'clogF' is the estimated cumulative hazard, and '_cons' refers to the constant term. '2._times#c._clogF' is the interaction term between time point 2 and the estimated cumulative hazard. | | | | | | |
