# Supplementary material for: Development and validation of a scoring system to predict mortality in patients hospitalized with COVID-19: A retrospective cohort study in two large hospitals in Ecuador
Source: PLoS One. 2023 Jul 17;18(7):e0288106. doi: 10.1371/journal.pone.0288106 (PMC10351692; doi:10.1371/journal.pone.0288106)
Supplement: S6 Table — (DOCX) [file pone.0288106.s007.docx]

**S6 Table. - Cox regression modelling with multiple imputations was applied.**

| The model was defined as follows: |
| --- |
| h(t\|x) = h0(t) * exp(b1 * sex + b2 * age in quartiles + b3 * hypoxemia + b4 * hospital glycaemia (categorical) + b5 * RCP (binomial) + b6 * pH (categorical) + b7 * AST:ALT ratio (categorical) + b8 * leucocytosis (categorical)) |
| where: |
| h(t\|x) represents the instantaneous hazard rate at time t, conditioned on the predictor variables x. |
| h0(t) is the baseline hazard function at time t. |
| b1, b2, ..., b8 are the estimated coefficients for each of the predictor variables. |
