# Supplementary material for: Development and validation of a scoring system to predict mortality in patients hospitalized with COVID-19: A retrospective cohort study in two large hospitals in Ecuador
Source: PLoS One. 2023 Jul 17;18(7):e0288106. doi: 10.1371/journal.pone.0288106 (PMC10351692; doi:10.1371/journal.pone.0288106)
Supplement: S7 Table — (DOCX) [file pone.0288106.s008.docx]

**S7 Table. - Comparison of Accuracy and Other Metrics between Guayaquil an Quito.**

| **City** | **Cutoff Point** | **Sensitivity** | **Specificity** | **LR+** | **LR-** | **Accuracy** |
| --- | --- | --- | --- | --- | --- | --- |
| Guayaquil | >= 65 | 23.80% | 91.43% | 2.776 | 0.833 | 63.87% |
| Quito | >= 66 | 8.86% | 99.20% | 11.12 | 0.919 | 88.36% |
| Note: Sensitivity represents the proportion of true positive cases correctly identified. Specificity denotes the proportion of true negative cases correctly identified. LR+ indicates the strength of association between the score and mortality risk, while LR- signifies the strength of negative association. Accuracy refers to the overall correct classification rate based on the chosen cutoff point. | | | | | | |
