# Supplementary material for: Development and validation of a scoring system to predict mortality in patients hospitalized with COVID-19: A retrospective cohort study in two large hospitals in Ecuador
Source: PLoS One. 2023 Jul 17;18(7):e0288106. doi: 10.1371/journal.pone.0288106 (PMC10351692; doi:10.1371/journal.pone.0288106)
Supplement: S8 Table — (DOCX) [file pone.0288106.s009.docx]

*S8 Table. Crude and adjusted associations between baseline variables at admission and in-hospital death, according to our parsimonious model on Table 3 of the main text (Cox proportional hazards model) stratified by sex (validation cohort).*

| **Variable** | **Adjusted parsimonious model *(see Table 3 of the main text)***  **HR (95% CI)** | | | |
| --- | --- | --- | --- | --- |
|  | **Women**  **n=928** | **p-value** | **Men**  **n=1569** | **p-value** |
| Age categories |  |  |  |  |
| *17 to 44 years old (ref.)* | 1 | - | 1 | - |
| *45 to 57 years old* | 1.59 (0.48 to 5.32) | 0.452 | 1.63 (0.91 to 2.92) | 0.100 |
| *58 to 68 years old* | 3.21 (0.84 to 12.29) | 0.089 | 2.59 (1.50 to 4.47) | 0.001 |
| *69 to 102 years old* | 4.20 (1.17 to 15.11) | 0.028 | 3.04 (1.82 to 5.09) | <0.001 |
| *p-for-trend* | 1.56 (1.19 to 2.05) | 0.001 | 1.40 (1.22 to 1.60) | <0.001 |
| Vital signs |  |  |  |  |
| *Hypoxia (no hypoxia is the ref.)****^a^*** | 1.67 (0.96 to 2.89) | 0.068 | 1.31 (0.90 to 1.91) | 0.162 |
| Laboratory parameters |  |  |  |  |
| *Glucose categories* |  |  |  |  |
| *70 to ≤140 mg/dL (ref.)* | 1 | - | 1 | - |
| *<70 mg/dL* | 1.21 (0.30 to 4.92) | 0.786 | 2.37 (1.18 to 4.75) | 0.015 |
| *>140 mg/dL* | 1.28 (0.76 to 2.15) | 0.357 | 1.27 (0.98 to 1.65) | 0.072 |
| *AST to ALT ratio >1 (otherwise is the ref.)****^c^*** | 1.66 (1.08 to 2.56) | 0.022 | 1.53 (1.21 to 1.93) | <0.001 |
| *C-reactive protein >10 mg/dL (otherwise is the ref.)* | 1.61 (0.97 to 2.67) | 0.068 | 1.44 (1.03 to 2.02) | 0.032 |
| *Arterial pH categories* |  |  |  |  |
| *7.35 to 7.45 (ref.)* | 1 | - | 1 | - |
| *<7.35* | 1.40 (0.90 to 2.18) | 0.140 | 1.37 (1.02 to 1.84) | 0.036 |
| *>7.45* | 0.86 (0.49 to 1.51) | 0.601 | 1.16 (0.87 to 1.56) | 0.307 |
| *White blood cell count* |  |  |  |  |
| *>10 x10^3^ per μL (otherwise is the ref.)* | 1.86 (1.12 to 3.10) | 0.016 | 1.71 (1.27 to 2.32) | <0.001 |
| HR = hazard ratios, 95% CI= 95% confidence interval, AST=Alanine transaminase.  ***^a^***=hypoxia was defined when oxygen saturation at admission was <95% in Guayaquil (0 meters above the sea level and <92% in Quito (2885 meters above the sea level). | | | | |
