# Supplementary material for: Development and validation of a scoring system to predict mortality in patients hospitalized with COVID-19: A retrospective cohort study in two large hospitals in Ecuador
Source: PLoS One. 2023 Jul 17;18(7):e0288106. doi: 10.1371/journal.pone.0288106 (PMC10351692; doi:10.1371/journal.pone.0288106)
Supplement: S9 Table — (DOCX) [file pone.0288106.s010.docx]

*S9 Table. Crude and adjusted associations between baseline variables at admission and in-hospital death, according to our parsimonious model on Table 3 of the main text (Cox proportional hazards model) excluding: (i) patients in both extreme of age quartiles, (ii) patients with ≥180 mmHg of systolic blood pressure or ≥90 mmHg of diastolic blood pressure; and (iii) patients with ≥200 mg/dL of serum glucose (validation cohort).*

|  | **Excluding those in younger quartile of age**  **n=1852** | **p-value** | **Excluding those in older quartile of age**  **n=1878** | **p-value** | **Excluding those with very high blood pressure (SBP ≥180 or DBP ≥120 mmHg)**  **n=2478** | **p-value** | **Excluding those with serum glucose ≥200 mg/dL**  **n=2218** | **p-value** |
| --- | --- | --- | --- | --- | --- | --- | --- | --- |
| Male sex (female is the ref.) | 1.31 (1.01 to 1.69) | 0.036 | 1.41 (0.99 to 2.02) | 0.059 | 1.32 (1.04 to 1.69) | 0.025 | 1.33 (1.02 to 1.74) | 0.036 |
| Age categories |  |  |  |  |  |  |  |  |
| *17 to 44 years old (ref.)* | Omitted | - | 1 | - | 1 | - | 1 | - |
| *45 to 57 years old* | 1 | - | 1.41 (0.99 to 2.02) | 0.059 | 1.58 (0.92 to 2.71) | 0.092 | 1.66 (0.92 to 3.00) | 0.092 |
| *58 to 68 years old* | 1.70 (1.19 to 2.41) | 0.005 | 2.25 (1.24 to 4.09) | 0.011 | 2.66 (1.50 to 4.72) | 0.002 | 2.72 (1.43 to 5.17) | 0.004 |
| *69 to 102 years old* | 2.07 (1.45 to 2.97) | <0.001 | Omitted | - | 3.26 (1.85 to 5.72) | <0.001 | 3.55 (1.89 to 6.68) | 0.001 |
| *p-for-trend* | 1.39 (1.18 to 1.65) | <0.001 | 1.52 (1.16 to 1.98) | 0.005 | 1.44 (1.25 to 1.66) | <0.001 | 1.49 (1.26 to 1.76) | <0.001 |
| Vital signs |  |  |  |  |  |  |  |  |
| *Hypoxia (no hypoxia is the ref.)a* | 1.32 (0.96 to 1.83) | 0.087 | 1.53 (0.98 to 2.39) | 0.062 | 1.40 (1.01 to 1.95 ) | 0.042 | 1.45 (1.01 to 2.08) | 0.047 |
| Laboratory parameters |  |  |  |  |  |  |  |  |
| *Glucose categories* |  |  |  |  |  |  |  |  |
| *70 to ≤140 mg/dL (ref.)* | 1 | - | 1 | - | 1 | - | 1 | - |
| *<70 mg/dL* | 1.99 (1.02 to 3.88) | 0.043 | 2.07 (0.96 to 4.47) | 0.062 | 2.00 (1.02 to 3.90) | 0.043 | 2.00 (1.01 to 3.96) | 0.048 |
| *>140 mg/dL* | 1.26 (0.98 to 1.62) | 0.067 | 1.49 (1.08 to 2.07) | 0.018 | 1.24 (0.96 to 1.61) | 0.094 | 1.16 (0.86 to 1.55) | 0.318 |
| *AST to ALT ratio >1 (otherwise is the ref.)c* | 1.54 (1.23 to 1.92) | <0.001 | 1.74 (1.30 to 2.32) | <0.001 | 1.57 (1.25 to 1.96) | <0.001 | 1.45 (1.13 to 1.86) | 0.004 |
| *C-reactive protein >10 mg/dL (otherwise is the ref.)* | 1.43 (1.02 to 2.00) | 0.040 | 1.57 (1.03 to 2.41) | 0.038 | 1.50 (1.07 to 2.11) | 0.021 | 1.55 (1.07 to 2.23) | 0.021 |
| *Arterial pH categories* |  |  |  |  |  |  |  |  |
| *7.35 to 7.45 (ref.)* | 1 | - | 1 | - | 1 | - | 1 | - |
| *<7.35* | 1.34 (1.02 to 1.77) | 0.036 | 1.52 (1.04 to 2.21) | 0.030 | 1.42 (1.08 to 1.86) | 0.014 | 1.44 (1.07 to 1.92) | 0.016 |
| *>7.45* | 1.07 (0.83 to 1.37) | 0.593 | 1.17 (0.81 to 1.70) | 0.399 | 1.09 (0.84 to 1.41) | 0.518 | 1.15 (0.86 to 1.55) | 0.343 |
| *White blood cell count* |  |  |  |  |  |  |  |  |
| *>10 x10^3^ per μL (otherwise is the ref.)* | 1.70 (1.32 to 2.20) | <0.001 | 2.08 (1.42 to 3.05) | 0.001 | 1.80 (1.38 to 2.34) | <0.001 | 1.78 (1.29 to 2.45) | 0.001 |
| HR = hazard ratios, 95% CI= 95% confidence interval, AST=Alanine transaminase.  ***^a^***=hypoxia was defined when oxygen saturation at admission was <95% in Guayaquil (0 meters above the sea level and <92% in Quito (2885 meters above the sea level). | | | | | | | | |
