# Supplementary material for: Development and validation of a scoring system to predict mortality in patients hospitalized with COVID-19: A retrospective cohort study in two large hospitals in Ecuador
Source: PLoS One. 2023 Jul 17;18(7):e0288106. doi: 10.1371/journal.pone.0288106 (PMC10351692; doi:10.1371/journal.pone.0288106)
Supplement: S10 Table — (DOCX) [file pone.0288106.s011.docx]

**S10 Table. -** **Comparative detailed report of sensitivity and specificity between Guayaquil and Quito.**

| **Detailed report of sensitivity and specificity for Guayaquil** | | | | | |
| --- | --- | --- | --- | --- | --- |
| **Cutpoint** | **Sensitivity** | **Specificity** | **Correctly Classified** | **LR+** | **LR-** |
| ( >= 35 ) | 94.55% | 29.91% | 56.25% | 1.3489 | 0.1823 |
| ( >= 36 ) | 93.59% | 31.77% | 56.96% | 1.3717 | 0.2017 |
| ( >= 37 ) | 93.20% | 33.06% | 57.56% | 1.3922 | 0.2058 |
| ( >= 38 ) | 92.43% | 35.15% | 58.49% | 1.4253 | 0.2154 |
| ( >= 39 ) | 91.82% | 36.96% | 59.31% | 1.4564 | 0.2214 |
| ( >= 40 ) | 90.65% | 39.32% | 60.24% | 1.4940 | 0.2377 |
| ( >= 41 ) | 89.52% | 42.16% | 61.46% | 1.5477 | 0.2487 |
| ( >= 42 ) | 86.71% | 45.42% | 62.24% | 1.5887 | 0.2926 |
| ( >= 43 ) | 85.78% | 47.90% | 63.33% | 1.6464 | 0.2968 |
| **Detailed report of sensitivity and specificity for Quito** | | | | | |
| **Cutpoint** | **Sensitivity** | **Specificity** | **Correctly Classified** | **LR+** | **LR-** |
| ( >= 35 ) | 88.23% | 61.04% | 64.20% | 2.2644 | 0.1929 |
| ( >= 36 ) | 87.26% | 63.33% | 66.12% | 2.3798 | 0.2012 |
| ( >= 37 ) | 86.12% | 64.46% | 66.98% | 2.4231 | 0.2153 |
| ( >= 38 ) | 83.86% | 66.61% | 68.61% | 2.5110 | 0.2424 |
| ( >= 39 ) | 82.78% | 67.75% | 69.50% | 2.5667 | 0.2542 |
| ( >= 40 ) | 79.43% | 70.52% | 71.55% | 2.6940 | 0.2917 |
| ( >= 41 ) | 78.02% | 71.65% | 72.39% | 2.7519 | 0.3067 |
| ( >= 42 ) | 75.16% | 75.96% | 75.87% | 3.1270 | 0.3270 |
| ( >= 43 ) | 74.19% | 77.18% | 76.83% | 3.2510 | 0.3344 |
