# Supplementary material for: Development and validation of a scoring system to predict mortality in patients hospitalized with COVID-19: A retrospective cohort study in two large hospitals in Ecuador
Source: PLoS One. 2023 Jul 17;18(7):e0288106. doi: 10.1371/journal.pone.0288106 (PMC10351692; doi:10.1371/journal.pone.0288106)
Supplement: S11 Table — (DOCX) [file pone.0288106.s012.docx]

**S11 Table. - Sensitivity, specificity, and classification accuracy of the proposed risk classification cutpoints.**

| Cutpoint | Sensitivity | Specificity | Correctly Classified | LR+ | LR- |
| --- | --- | --- | --- | --- | --- |
| <= 30 | >96.55% | <51.41% | <56.12% | <1.9869 | >0.0671 |
| 31-65 | 96.55% to 20.69% | 56.22% to 98.80% | 60.43% to 90.65% | 2.2056 to 17.1724 | 0.0613 to 0.8028 |
| > 65 | <20.69% | > 98.80% | >90.65% | >17.1724 | <0.8028 |
