# Supplementary material for: Development and validation of a scoring system to predict mortality in patients hospitalized with COVID-19: A retrospective cohort study in two large hospitals in Ecuador
Source: PLoS One. 2023 Jul 17;18(7):e0288106. doi: 10.1371/journal.pone.0288106 (PMC10351692; doi:10.1371/journal.pone.0288106)
Supplement: Attachment — Submitted filename: Reviewer recommendations.docx.pdf [file pone.0288106.s014.pdf]

Development and validation of a scoring system to predict mortality in patients hospitalized with COVID-19: a retrospective cohort study in two large hospitals in Ecuador

Reviewer's recommendations

This document is considered to be of good quality and that it provides a lot of relevant information to understand and, above all, consider certain aspects in the care of COVID patients. Some small observations are presented in order to improve the contribution of the proposed article.

"At present, despite the two-dose vaccination prevalence being close to 77.87 % (one of the highest in the region) [12,16], there are difficulties reaching populations where vaccination coverage is complex (i.e., rural areas population). In that sense, a notable increase in cases has been reported during January 2022 [7]."

It is unlikely that the only and main cause of the increase in cases is due to vaccination coverage, it would be important to analyze the possible impact of the mobilization of the population for vacation periods or other festivities that impacted the increase in cases, relaxation of protection measures, distancing in the population, restart of economic activities, increase in capacity in establishments, etc.

The groups analyzed should also be considered with the issue of the height of the sea level, considering that one city is above 2,800 meters and the other city is at sea level. This could be a factor that should be taken into account when analyzing and comparing the results. It would be interesting to have the analysis by cities. Some parameters analyzed could have an impact for the simple reason of the altitude in which they are living.
