# Supplementary material for: Development and validation of a scoring system to predict mortality in patients hospitalized with COVID-19: A retrospective cohort study in two large hospitals in Ecuador
Source: PLoS One. 2023 Jul 17;18(7):e0288106. doi: 10.1371/journal.pone.0288106 (PMC10351692; doi:10.1371/journal.pone.0288106)
Supplement: Attachment — Submitted filename: Reviewer recommendations.pdf [file pone.0288106.s016.pdf]

Development and validation of a scoring system to predict mortality in patients hospitalized with COVID-19: a retrospective cohort study in two large hospitals in Ecuador

Reviewer's recommendations

The document sent with the changes made has improved compared to its previous version, With the new changes made in the document it is considered that it is ready for publication. Congratulations to the authors
