# Supplementary material for: Development and validation of a scoring system to predict mortality in patients hospitalized with COVID-19: A retrospective cohort study in two large hospitals in Ecuador
Source: PLoS One. 2023 Jul 17;18(7):e0288106. doi: 10.1371/journal.pone.0288106 (PMC10351692; doi:10.1371/journal.pone.0288106)
Supplement: Attachment — Submitted filename: Rebuttal letter_24022023.docx [file pone.0288106.s015.docx]

**Rebuttal letter to reviewers’ comments to authors**

**PLOS ONE**

**Manuscript ID:** PONE-D-22-16810

**Title:** Development and validation of a scoring system to predict mortality in patients hospitalized with COVID-19: a retrospective cohort study in two large hospitals in Ecuador

**Authors:** Iván Dueñas-Espín, María Echeverría-Mora, Camila Montenegro-Fárez, Manuel Baldeón, Luis Chantong Villacres, Hugo Espejo Cárdenas, Marco Fornasini, Miguel Ochoa Andrade, Carlos Solís.

**Decision:** Revision required

**Article Type:** original article.

**Corresponding Author:** Iván Dueñas-Espín

We thank the Editor and Reviewers for their insightful comments that have been helpful to improve the manuscript. These comments are very much appreciated. This retrospective cohort study generated a system scoring model to predict the mortality of hospitalized COVID-19 patients at two large hospitals in Ecuador. This model found a higher COVID-19 mortality risk for males, patients of increasing age, patients with hypoxia, hypoglycemia, hyperglycemia, an AST to ALT ratio >1, elevated C-reactive protein, altered arterial pH, and leucocytosis. We believe that our study makes a significant contribution to the literature because the findings of this study are consistent with previous research, but some of the limitations of the previous studies were overcome in our study. Further, we believe that this paper will be of interest to the readership of your journal because COVID-19 is still a major problem globally, and this model could be used by physicians to treat patients at higher risk of mortality from COVID-19, therefore saving lives. We hope that our answers satisfy the questions and comments from the Editorial team and the reviewers.

**Dear Dr. Dueñas-Espín,**

**Thank you for submitting your manuscript to PLOS ONE. After careful consideration, we feel that it has merit but does not fully meet PLOS ONE’s publication criteria as it currently stands. Therefore, we invite you to submit a revised version of the manuscript that addresses the points raised during the review process.**

==============================
**Thanks for your patience, it has been difficult to find reviewers and as you can see on the comments there was quite a lot of discrepancy amongst them. As two of them were quite positive we would like you to address all the comments, especially those that are associated with the reviewer that proposed to reject the manuscript. The issues raised by that reviewer are important and we would like to see a revised version of the manuscript before we can further assess its suitability.**

**ANSWER:**

Thanks a lot for the insightful comments that reviewers and editorial team have done to our manuscript. We believe that, after the revision, we have answered properly all the comments in order to achieve suitability for being published in your prestigious journal.

**JOURNAL REQUIREMENTS:**

**When submitting your revision, we need you to address these additional requirements.**

**1. Please ensure that your manuscript meets PLOS ONE's style requirements, including those for file naming. The PLOS ONE style templates can be found at**

**https://journals.plos.org/plosone/s/file?id=wjVg/PLOSOne_formatting_sample_main_body.pdf and**

[**https://journals.plos.org/plosone/s/fileid=ba62/PLOSOne_formatting_sample_title_authors_affiliations.pdf**](https://journals.plos.org/plosone/s/fileid=ba62/PLOSOne_formatting_sample_title_authors_affiliations.pdf)**.**

**ANSWER:**

We have reviewed the guidelines and changed the manuscript accordingly. Please see marked and unmarked copies of the manuscript.

**2. In your Data Availability statement, you have not specified where the minimal data set underlying the results described in your manuscript can be found. PLOS defines a study's minimal data set as the underlying data used to reach the conclusions drawn in the manuscript and any additional data required to replicate the reported study findings in their entirety. All PLOS journals require that the minimal data set be made fully available. For more information about our data policy, please see http://journals.plos.org/plosone/s/data-availability.**

**Upon re-submitting your revised manuscript, please upload your study’s minimal underlying data set as either Supporting Information files or to a stable, public repository and include the relevant URLs, DOIs, or accession numbers within your revised cover letter. For a list of acceptable repositories, please see http://journals.plos.org/plosone/s/data-availability#loc-recommended-repositories. Any potentially identifying patient information must be fully anonymized.**

**Important: If there are ethical or legal restrictions to sharing your data publicly, please explain these restrictions in detail. Please see our guidelines for more information on what we consider unacceptable restrictions to publicly sharing data: http://journals.plos.org/plosone/s/data-availability#loc-unacceptable-data-access-restrictions. Note that it is not acceptable for the authors to be the sole named individuals responsible for ensuring data access.**

**We will update your Data Availability statement to reflect the information you provide in your cover letter.**

**3. We note that you have indicated that data from this study are available upon request. PLOS only allows data to be available upon request if there are legal or ethical restrictions on sharing data publicly. For more information on unacceptable data access restrictions, please see http://journals.plos.org/plosone/s/data-availability#loc-unacceptable-data-access-restrictions.**

**In your revised cover letter, please address the following prompts:**

**a) If there are ethical or legal restrictions on sharing a de-identified data set, please explain them in detail (e.g., data contain potentially sensitive information, data are owned by a third-party organization, etc.) and who has imposed them (e.g., an ethics committee). Please also provide contact information for a data access committee, ethics committee, or other institutional body to which data requests may be sent.**

**b) If there are no restrictions, please upload the minimal anonymized data set necessary to replicate your study findings as either Supporting Information files or to a stable, public repository and provide us with the relevant URLs, DOIs, or accession numbers. For a list of acceptable repositories, please see http://journals.plos.org/plosone/s/data-availability#loc-recommended-repositories.**

**We will update your Data Availability statement on your behalf to reflect the information you provide.**

**ANSWER:**

Thank you very much. After a brief discussion with the authors of the manuscript we decided to make the data public for proper replication of our results. We have changed the “Data Availability” statement in this way: “All relevant data are within the manuscript and its Supporting Information files”. Therefore, we share the database as a supporting information file “S1 Data” as a “.csv” file. In that regard, we have changed the Data Availability statement as follows:

“Data Availability Statement: All relevant data are within the manuscript and its Supporting information files.”

This new statement has been mentioned in the new covering letter.

**COMMENTS TO THE AUTHOR**

**1. Is the manuscript technically sound, and do the data support the conclusions?

The manuscript must describe a technically sound piece of scientific research with data that supports the conclusions. Experiments must have been conducted rigorously, with appropriate controls, replication, and sample sizes. The conclusions must be drawn appropriately based on the data presented.**

**Reviewer #1: Yes**

**Reviewer #2: Partly**

**Reviewer #3: Yes**

**ANSWER:**

Thank you for your positive feedback, we have clarified and completed several parts of the main text and supplementary material to improve the coherence along the whole manuscript.

**2. Has the statistical analysis been performed appropriately and rigorously?**

**Reviewer #1: Yes**

**Reviewer #2: Yes**

**Reviewer #3: Yes**

**ANSWER:**

Thank you for your positive feedback.

**3. Have the authors made all data underlying the findings in their manuscript fully available?

The** [**PLOS Data policy**](http://www.plosone.org/static/policies.action#sharing) **requires authors to make all data underlying the findings described in their manuscript fully available without restriction, with rare exception (please refer to the Data Availability Statement in the manuscript PDF file). The data should be provided as part of the manuscript or its supporting information, or deposited to a public repository. For example, in addition to summary statistics, the data points behind means, medians and variance measures should be available. If there are restrictions on publicly sharing data—e.g. participant privacy or use of data from a third party—those must be specified.**

**Reviewer #1: No**

**Reviewer #2: Yes**

**Reviewer #3: Yes**

**ANSWER:**

Thank you for your positive feedback. We have modified the Data Availability Statement. Now, we share the database as a supporting information file “S1 Data” as a “.csv” file.

**4. Is the manuscript presented in an intelligible fashion and written in standard English?

PLOS ONE does not copyedit accepted manuscripts, so the language in submitted articles must be clear, correct, and unambiguous. Any typographical or grammatical errors should be corrected at revision, so please note any specific errors here.**

**Reviewer #1: Yes**

**Reviewer #2: Yes**

**Reviewer #3: Yes**

**ANSWER:**

Thank you for your positive feedback.

**5. Review Comments to the Author**

**Please use the space provided to explain your answers to the questions above. You may also include additional comments for the author, including concerns about dual publication, research ethics, or publication ethics. (Please upload your review as an attachment if it exceeds 20,000 characters)**

**ANSWERS TO REVIEWERS**

**ANSWERS TO REVIEWER #1:**

**Reviewer #1: This document is considered to be of good quality and that it provides a lot of relevant information to understand and, above all, consider certain aspects in the care of COVID patients. Some small observations are presented in order to improve the contribution of the proposed article.**

**ANSWER:**

Thanks a lot for your important feedback. We have carefully read your comments and we have changed the manuscript according to satisfy them.

***“At present, despite the two-dose vaccination prevalence being close to 77.87 % (one of the highest in the region) [12,16], there are difficulties reaching populations where vaccination coverage is complex (i.e., rural areas population). In that sense, a notable increase in cases has been reported during January 2022 [7].”***

**It is unlikely that the only and main cause of the increase in cases is due to vaccination coverage, it would be important to analyze the possible impact of the mobilization of the population for vacation periods or other festivities that impacted the increase in cases, relaxation of protection measures, distancing in the population, restart of economic activities, increase in capacity in establishments, etc.**

**ANSWER:**

Tanks a lot for your important observation. We have reviewed literature and concluded the same as you. The factors which affect negatively for a further dissemination of the disease goes far away than the simple lack of vaccination coverage. In that regard we have added next text in the “Introduction” section, lines 96 to 111, page 6 and 7, as follows:

“Among the different possible explanations, the following aspects are the ones that could mainly explain it: *(i)* vaccines are not 100% effective, *(ii)* unvaccinated people could be fueling transmission, *(iii)* spreading the disease by vaccinated cases, *(iv)* people that are moving around more to restart economic activities or during vacation periods and festivities, *(v)* the relaxation of protection measures like distancing measures or the increase in capacity in establishments; and *(vi)* the current global dominance of the SARS-CoV-2 Omicron (BA.1 or B.1.1.529) and several subvariants that are appearing more frequently, with a higher transmissibility, even in vaccinated individuals (1)…”

**References:**

1. Khandia, R., Singhal, S., Alqahtani, T., Kamal, M. A., El-Shall, N. A., Nainu, F., Desingu, P. A., & Dhama, K. (2022). Emergence of SARS-CoV-2 Omicron (B.1.1.529) variant, salient features, high global health concerns and strategies to counter it amid ongoing COVID-19 pandemic. In Environmental Research (Vol. 209). Academic Press Inc. <https://doi.org/10.1016/j.envres.2022.112816>.
2. Hyams, C., Challen, R., Marlow, R., Nguyen, J., Begier, E., Southern, J., King, J., Morley, A., Kinney, J., Clout, M., Oliver, J., Gray, S., Ellsbury, G., Maskell, N., Jodar, L., Gessner, B., McLaughlin, J., Danon, L., Finn, A., … Szasz-Benczur, Z. (2023). Severity of Omicron (B.1.1.529) and Delta (B.1.617.2) SARS-CoV-2 infection among hospitalised adults: A prospective cohort study in Bristol, United Kingdom. The Lancet Regional Health - Europe, 25, 1–11. <https://doi.org/10.1016/j.lanepe.2022.100556>.

**The groups analyzed should also be considered with the issue of the height of the sea level, considering that one city is above 2,800 meters and the other city is at sea level. This could be a factor that should be taken into account when analysing and comparing the results. It would be interesting to have the analysis by cities. Some parameters analyzed could have an impact for the simple reason of the altitude in which they are living.**

**ANSWER:**

We thank the reviewer. Your comment has been of great value to enrich the content of our manuscript. We have to mention that, indeed, it was planned to perform the analyses of the discriminatory power of the score between hospitals and between sex categories, calculating the Area under the ROC curve in the validation cohort. We kindly apologize for the omission. In that regard, we show the results in the new **S2 Table. “**Comparison of the discriminatory power of the score between hospitals and between sex categories, calculating the Area under the ROC curve in the validation cohort.” The order of the other supplementary tables were changed according to the insertion of the new table. In that sense, we modified the text at line 214 of page 11 of the “Material and Methods” section adding:

“We compared the discriminatory power of the score between hospitals and between sex categories, calculating the AUC in the two hospitals and both sex categories (**S2 Table**)”.

Furthermore, in the “Results” section (first paragraph of 19^th^ page), in which added a text as follows:

“The discriminative ability when tested the score in the validation cohort by hospitals and sex categories showed less discriminative capacity when the stratified analyses were performed for the categories of the hospital and sex variables (**S2 Table**), especially when the analysis was performed in Guayaquil hospital (AUC of the ROC curve of 0.742, 95% CI: 0.712 to 0.773) and when it was performed in females (AUC of the ROC curve of 0.829, 95% CI: 0.671 to 0.986).”

Finally, at the light of those findings, we added a text in the “Discussion” section, in the last paragraph of the page 20, as follows:

“Nevertheless, when we circumscribed the calculation of the AUC to female hospitalized patients and to patients of the Guayaquil hospital, the AUC was reduced. These findings could be explained by several factors: *(i)* the score could be less effective in predicting in-hospital death at the sea level, maybe the lower COVID-19 related deaths in altitude were the results from a biological adaptation to hypoxia, due to environmental factors, (3) or related to the different contexts between both cities/altitudes (4), *(ii)* the score could be less effective in predicting in-hospital death for females maybe because of the lack of information regarding comorbidities which are, generally, more frequent among females (5), and *(iii)* the lack of a similar representativeness of Guayaquil and female population by comparison with their counterparts (patients from Quito and male), when building the score. In summary, the score seems to work best for hospitalized men in Quito.”

**References:**

3. Simbaña-Rivera K, Jaramillo PRM, Silva JVV, Gómez-Barreno L, Campoverde ABV, Novillo Cevallos JF, et al. High-altitude is associated with better short-term survival in critically ill COVID-19 patients admitted to the ICU. PLoS One. 2022;17(3):e0262423.
4. Abbasi BA, Chanana N, Palmo T, Pasha Q. Disparities in COVID-19 incidence and fatality rates at high-altitude. 2023;2:1–16.
5. Gu H, Wang C, Zhang X, Jiang Y, Li H, Meng X. Ten-Year Trends in Sex Differences in Cardiovascular Risk Factors, In-Hospital Management, and Outcomes of Ischemic Stroke in China: Analyses of a Nationwide Serial Cross-Sectional Survey from 2005 to 2015. Int J Stroke. 2023;Feb 8:1747(Epub ahead of print):2023.

**ANSWERS TO REVIEWER 2**

**Reviewer #2: In their manuscript, the authors build a risk scoring system for mortality during hospitalization for COVID-19. While the paper is of interest, there are some major technical issues that have to be addressed.**

**Major comments:**

**Not having the ability to include the other clinical information- mechanical ventilation and drug/antibody usage in the risk scoring system is a major limitation and has to be included as those are two of the most common interventions and have shown to have predictive value in recovery.**

**ANSWER:**

Thanks a lot for your accurate observation, it has been very useful for improving the manuscript. Unfortunately, the database did not include information about other clinical information as the use of mechanical ventilation nor the use of monoclonal antibodies; therefore, it is impossible to test those variables to assess if the discriminatory ability of the score improves. In any case, we looked for other possibilities to try to explore the potential effect of these important variables.

Regarding the mechanical ventilation usage, evidence shows that the use of mechanical ventilation is very associated with a high mortality in patients with COVID-19; nevertheless, it is important to note that this factor is usually evaluated as an outcome (6) Given the extremely high mortality rate among ventilated patients (10) it is not expected to use this variable as a predictor of intrahospital mortality, especially when it is expected to use the score very early after the admission, for taking timely clincal decisions.

Despite that, and after a brief revision of the literature to identify potential predictors related with the necessity of mechanical ventilation of the most relevant scores and predictive tools of hospital mortality in COVID-19 patients, we found the ROX-index as a potential predictor into the score for predicting in-hospital mortality (11). In that regard, as we proceeded with the rest of other potential predictors, we first calculated the hazard ratio per each increase in the ROX-index score by including the index in both, the saturated Cox regression model [Hazard ratio (HR)=1.00; 95% Confidence Interval(95%CI): 0.96 to 1.05], and in the parsimonious model (HR= 0.98; 95%CI: 0.96 to 1.01). Unfortunately, the ROX score did not added statistically significant information and it was not included in a subsequent score.

Since the intention of this study was to construct a scoring system for the risk of in-hospital mortality that is useful for predicting mortality from the first moment of admission to the hospital, and since many times the requirement for mechanical ventilation , while tracheal intubation for mechanical ventilation occurred within 7 days after admission (12) it seems that including the predictor is not very useful in a score of being applied at the moment of the admission to the hospital. In addition, to our knowledge, there are no risk scoring systems that use the use of mechanical ventilation as a predictor of in-hospital mortality.

In any case, recognizing the interesting observation from the reviewer, we have constructed the variable “respiratory failure” when two or more of the following criteria were present: (i) respiratory rate >20 or <10, (ii) pCO2 > 50 mmHg & pH <7.35, or (iii) pO2 < 50 mmHg for Quito patients or pO2 < 60 mmHg for Guayaquil patients. After running the models (with and without the respiratory failure variable) we created two scores and calculated the ROC AUC being higher in the score without the “respiratory failure” variable (0.827, (95%CI: 0.810 to 0.8455) vs. 0.817 (95%CI: 0.798 to 0.836)). Thus, we did not continue to the next steps in building the score using this variable.

Regarding the use of drugs as the monoclonal antibodies we reviewed if any of the patients received them and corroborated that none of the patients received monoclonal antibodies. There is not enough evidence of other drugs could be useful for reducing in-hospital mortality in COVID-19 patients, except for specific monoclonal antibodies. We performed a brief searching for scores with monoclonal antibodies bye the next strategy (monoclonal antibodies as a predictor AND risk score AND COVID-19) and did not find papers about a score of in-hospital mortality including use of monoclonal antibodies. But what we found was that a *“… score could assist clinicians in identifying, early after tocilizumab administration, patients who are likely to progress to mechanical ventilation or death, so that they could be selected for eventual rescue therapies”* which is poorly useful in contexts as Ecuadorian one in which the use of those agents is quite infrequent.

Moreover, we reviewed the most important papers regarding the use of monoclonal antibodies in the treatment of hospitalized patients with COVID-19 and its prognosis, furthermore, we briefly requested to the clinicians of our researchers’ team and colleagues from public and private hospitals in Quito-Ecuador and corroborated that any hospital in public sector of Ecuador is using any of the next antibodies: combination of casirivimab/imdevimab or tocilizumab. In that regard, we believe that, despite the usage of those medications is useful to improve the prognosis and, therefore, has significant value in predicting hospital mortality, their usage could not be realistically used in the prediction model. We did not include any medication in the score given that any of the papers reviewed included medications, other than the abovementioned monoclonal antibodies, as a predictor.

Regarding the usage of antibodies against SARS-CoV-2, we found papers which demonstrate that such antibodies could be useful for estimating prognosis in COVID-19 hospitalized patients. Nevertheless, the evidence is controversial, as some studies suggested that higher positive antibodies against SARS-CoV-2 (IgG or IgM) could be protector against more severe disease, other studies suggested that negative or lower titters of antibodies could be protector against more severe disease. In any case, we did not take into account the titters of antibodies against SARS-CoV-2 given that the methodology for measuring neutralizing antibodies was not properly developed as early to February to July 2020, when the participants were admitted to the hospital. We apologize for the lack of such important information. In any case, we consider that the lack of such information, or the lack of such predictors into the score we proposed, it is not a significant source of bias or dismisses the validity of our score. The reasons are: *(i)* the predictive ability of the antibodies is modest in most studies, *(ii)* the usage of antibody titters for predicting severity in COVID-19 has controversial directionality, it means that, on the one hand, the negative results have been associated with higher in-hospital mortality; for the other hand, the negative results have been associated with lower in-hospital mortality. To our knowledge, the association between neutralizing antibody titters and mortality must be elucidated first, before testing this variable as a potential predictor into a score of in hospital mortality for COVID-19.

Our intention was not to identify the predictive power of the use of mechanical ventilation for in-hospital mortality since COVID-19 mortality is extremely high when the disease is severe (7), several studies have shown that non-survivors required non-invasive (continuous positive airway pressure and biphasic positive airway pressure modes) or invasive ventilation more frequently than survivors (13), but rather detect early during hospitalization, patients at high risk of death.

**References:**

6. Kabbaha S, Al-Azzam S, Karasneh RA, Khassawneh BY, Al-Mistarehi AH, Lattyak WJ, et al. Predictors of invasive mechanical ventilation in hospitalized COVID-19 patients: a retrospective study from Jordan. Expert Rev Respir Med [Internet]. 2022;16(8):945–52. Available from: https://doi.org/10.1080/17476348.2022.2108796
7. Yang X, Yu Y, Xu J, Shu H, Xia J, Liu H, et al. Clinical course and outcomes of critically ill patients with SARS-CoV-2 pneumonia in Wuhan, China: a single-centered, retrospective, observational study. Lancet Respir Med [Internet]. 2020;8(5):475–81. Available from: <http://dx.doi.org/10.1016/S2213-2600(20)30079-5>
8. Petrilli CM, Jones SA, Yang J, Rajagopalan H, O’Donnell L, Chernyak Y, et al. Factors associated with hospital admission and critical illness among 5279 people with coronavirus disease 2019 in New York City: Prospective cohort study. BMJ. 2020;369.
9. Kim L, Garg S, O’Halloran A, Whitaker M, Pham H, Anderson EJ, et al. Risk Factors for Intensive Care Unit Admission and In-hospital Mortality among Hospitalized Adults Identified through the US Coronavirus Disease 2019 (COVID-19)-Associated Hospitalization Surveillance Network (COVID-NET). Clin Infect Dis. 2021;72(9):E206–14.
10. Barman Roy D, Gupta V, Tomar S, Gupta G, Biswas A, Ranjan P, et al. Epidemiology and Risk Factors of COVID-19-Related Mortality. Cureus. 2021;13(12):1–8.
11. Roca, O., Messika, J., Caralt, B., García-de-Acilu, M., Sztrymf, B., Ricard, J. D., & Masclans, J. R. (2016). Predicting success of high-flow nasal cannula in pneumonia patients with hypoxemic respiratory failure: The utility of the ROX index. Journal of Critical Care, 35, 200–205. https://doi.org/10.1016/j.jcrc.2016.05.022
12. Wargny M, Potier L, Gourdy P, Pichelin M, Amadou C, Benhamou PY, et al. Predictors of hospital discharge and mortality in patients with diabetes and COVID-19: updated results from the nationwide CORONADO study. Diabetologia. 2021;64(4):778–94.
13. Fumagalli C, Rozzini R, Vannini M, Coccia F, Cesaroni G, Mazzeo F, et al. Clinical risk score to predict in-hospital mortality in COVID-19 patients: a retrospective cohort study. BMJ Open. 2020;10(9):e040729.

**Minor comments:**

**Introduction- numbers for wordwide coronavirus statistics need to be updated,**

**ANSWER:**

Thanks a lot for your comments. In the “Introduction” section, lines 67 to 70 of the 5^th^ page, we have updated the COVID-19 statistics as follows:

“Currently, and despite that 69.7% of the world population has received at least one dose of a COVID-19 vaccine, 13.31 billion doses have been administered globally, and 954,258 are now administered each day, only 27.7% of people in low-income countries have received at least one dose (20)”

**Maybe mention which wave of variant caused the most hospitalizations
Omicron is mentioned but 2022 supporting omicron’s severity is not included**

**ANSWER:**

Thanks a lot for your comments. In the “Introduction” section, lines 72 to 74 of the 5^th^ page, we have updated the COVID-19 statistics as follows:

“Specifically, despite Delta variant caused more severe, the extremely contagious Omicron variant has resulted in a new collapse of public and private healthcare facilities increasing the risk for morbidity and mortality of the population.”

Furthermore, we have changed the “Introduction”, lines 92 to 111, pages 6^th^ and 7^th^, as follows:

“At present, despite the Ecuadorian prevalence of compliance with the complete vaccination protocol is being close to 79.06% (one of the highest in the region) (14,15,16), there are difficulties reaching populations where vaccination coverage is complex (i.e., rural areas population), a notable increase in cases has been reported during January 2022 (17). Among the different possible explanations, the following aspects are the ones that could mainly explain it: *(i)* vaccines are not 100% effective, *(ii)* unvaccinated people could be fueling transmission, *(iii)* spreading the disease by vaccinated cases, *(iv)* people that are moving around more to restart economic activities or during vacation periods and festivities, *(v)* the relaxation of protection measures like distancing measures or the increase in capacity in establishments; and *(vi)* the current global dominance of the SARS-CoV-2 Omicron (BA.1 or B.1.1.529) and several subvariants that are appearing more frequently, with a higher transmissibility, even in vaccinated individuals (18).”

**References:**

14. Boletines epidemiológicos coronavirus por semanas – Ministerio de Salud Pública [Internet]. [cited 2022 Mar 27]. Available from: <https://www.salud.gob.ec/boletines-epidemiologicos-coronavirus-por-semanas/>
15. Statista. COVID-19: dosis y porcentaje de vacunados por país de América Latina y el Caribe | Statista [Internet]. Porcentaje de vacunados y dosis. 2021 [cited 2022 May 17]. Available from: <https://es.statista.com/estadisticas/1258801/porcentaje-y-numero-vacunados-contra-covid-19-en-latinoamerica-por-pais>
16. Hasell J, Ortiz-Ospina E, Ritchie H, Roser M. “Coronavirus Pandemic (COVID-19)”. Our World in Data. In: https://ourworldindata.org/coronavirus [Internet]. 2022 [cited 2022 Mar 27]. Available from: <https://ourworldindata.org/coronavirus>
17. World Health Organization. WHO Coronavirus Disease (COVID-19) Dashboard With Vaccination Data | WHO Coronavirus (COVID-19) Dashboard With Vaccination Data [Internet]. World Health Organization. 2021 [cited 2022 May 17]. p. 1–5. Available from: <https://covid19.who.int/>
18. Khandia R, Singhal S, Alqahtani T, Kamal MA, El-Shall NA, Nainu F, et al. Emergence of SARS-CoV-2 Omicron (B.1.1.529) variant, salient features, high global health concerns and strategies to counter it amid ongoing COVID-19 pandemic. Vol. 209, Environmental Research. Academic Press Inc.; 2022.

**Unavailability of testing- is this in reference to early in the pandemic? This statement may need further clarification.**

**ANSWER:**

Thanks. We have changed the next paragraph of the “Introduction” section (lines 80 to 84 of the 6^th^ page:

In addition, it is important to consider that the rise of new variants is causing impacts in terms of public health that have not been seen since 2020 ~~because of the high rate of infection among healthcare workers, the inadequacy and unavailability of reliable testing, and unreliable data about reported cases~~ (19). These variants have increased the number of hospitalized patients throughout the region, and specifically in Ecuadorian territory.

As follows:

“In addition, it is important to consider that the rise of new variants is causing impacts in terms of public health that have not been seen since 2020 (19). These variants have increased the number of hospitalized patients throughout the region, and specifically in Ecuadorian territory.”

**Reference:**

19. Le Rutte EA, Shattock AJ, Chitnis Phd N, Kelly SL, Penny Phd MA. Assessing impact of Omicron on SARS-CoV-2 dynamics and public health burden. medRxiv [Internet]. 2021 Dec 14 [cited 2022 Mar 27];2021.12.12.21267673. Available from: https://www.medrxiv.org/content/10.1101/2021.12.12.21267673v1

**Percentage of booster uptake currently?**

**ANSWER:**

Thank you. As we mentioned above, In the “Introduction” section, lines 67 to 70 of the 5^th^ page, we have updated the COVID-19 statistics as follows:

“Currently, and despite that 69.7% of the world population has received at least one dose of a COVID-19 vaccine, 13.31 billion doses have been administered globally, and 954,258 are now administered each day, only 27.7% of people in low-income countries have received at least one dose (20)”

**References:**

20. Hasell J, Ortiz-Ospina E, Ritchie H, Roser M. “Coronavirus Pandemic (COVID-19)”. Our World in Data. In: https://ourworldindata.org/coronavirus [Internet]. 2022 [cited 2022 Mar 27]. Available from: <https://ourworldindata.org/coronavirus>.

**Conclusions-Elevated RT-PCR- what does that even mean? Higher viral load? Only can make that correlation if PCR is done with standard curve, unclear in methods**

**ANSWER:**

Thank you for your observation. We apologize for the mistake; in conclusions the proper acronym would be C-reactive protein (CPR). We have corrected it in several parts of the manuscript as you can corroborate in the new version of the manuscript.

**Reviewer #3: This manuscript by Dueñas-Espín et al described a retrospective study. One major limitation of the study is a large (28.6%) percentage of missing data. However the authors are transparent about the limitation, have used randomized methods to impute the missing data and have performed the analysis properly.**

Thank you very much.

**The manuscript is well-written. Even though findings are not surprinsing, it is consistent with what is expected from clinical studies of COVID-19 patients.**

Thank you very much.

**Despite the limitations, the authors should be commended for such a large study that includes thousands of patients.**

**ANSWER:**

Thanks a lot, we really appreciate your comment as we improved substantially the manuscript after your revision and the other reviewers’ comments.

**I think the authors do need to make some changes to make the manuscript better. Introduction section of the manuscript needs to be updated throughly with more recent statistics and appropirate references.**

**ANSWER:**

Thank you very much. In the “Introduction” section, lines 67 to 70 of the 5^th^ page, we have updated the COVID-19 statistics as follows:

“Currently, and despite that 69.7% of the world population has received at least one dose of a COVID-19 vaccine, 13.31 billion doses have been administered globally, and 954,258 are now administered each day, only 27.7% of people in low-income countries have received at least one dose (20)”

**In the abstract, include the abbreviation of C-reactive protein (CRP) and change "elevated PCR" to "elevated** **CRP".**

**ANSWER:**

Thanks a lot. Done.

**Result section, "2,565 patients are shown in S2 Table" should be "****2,565 patients in the validation cohort are shown in S2 Table"**

**ANSWER:**

Thank you very much, we did not have noticed the mistake before, we intended to mention that the biomarker was C-reactive protein (CRP) and not RT-PCR, in that regard, we have changed the text in the abstract and in different parts of the main text as it could be seen in the marked copy of the revised manuscript. Regarding the mistake in text, we apologize for that. We have corrected as you suggested.

**6. PLOS authors have the option to publish the peer review history of their article (**[**what does this mean?**](https://journals.plos.org/plosone/s/editorial-and-peer-review-process#loc-peer-review-history)**). If published, this will include your full peer review and any attached files.


If you choose “no”, your identity will remain anonymous but your review may still be made public.


Do you want your identity to be public for this peer review? For information about this choice, including consent withdrawal, please see our** [**Privacy Policy**](https://www.plos.org/privacy-policy)**.**

**Reviewer #1: No**

**Reviewer #2: No**

**Reviewer #3: No**

**[NOTE: If reviewer comments were submitted as an attachment file, they will be attached to this email and accessible via the submission site. Please log into your account, locate the manuscript record, and check for the action link "View Attachments". If this link does not appear, there are no attachment files.]**

**ANSWER:**

Thank you, we have considered all the comments we found in the attachment file.

**While revising your submission, please upload your figure files to the Preflight Analysis and Conversion Engine (PACE) digital diagnostic tool,** [**https://pacev2.apexcovantage.com/**](https://pacev2.apexcovantage.com/)**. PACE helps ensure that figures meet PLOS requirements. To use PACE, you must first register as a user. Registration is free. Then, login and navigate to the UPLOAD tab, where you will find detailed instructions on how to use the tool. If you encounter any issues or have any questions when using PACE, please email PLOS at** [**figures@plos.org**](mailto:figures@plos.org)**. Please note that Supporting Information files do not need this step.**

**ANSWER:**

Thank you very much for the comment. We have modified the figure using the PACE digital diagnostic tool.
