# Supplementary material for: Development and validation of a scoring system to predict mortality in patients hospitalized with COVID-19: A retrospective cohort study in two large hospitals in Ecuador
Source: PLoS One. 2023 Jul 17;18(7):e0288106. doi: 10.1371/journal.pone.0288106 (PMC10351692; doi:10.1371/journal.pone.0288106)
Supplement: Attachment — Submitted filename: Rebuttal letter_16062023.docx [file pone.0288106.s017.docx]

**Rebuttal letter to reviewers’ comments to editor**

**PLOS ONE**

**Manuscript ID:** PONE-D-22-16810R1

**Title:** Development and validation of a scoring system to predict mortality in patients hospitalized with COVID-19: a retrospective cohort study in two large hospitals in Ecuador

**Authors:** Iván Dueñas-Espín, María Echeverría-Mora, Camila Montenegro-Fárez, Manuel Baldeón, Luis Chantong Villacres, Hugo Espejo Cárdenas, Marco Fornasini, Miguel Ochoa Andrade, Carlos Solís.

**Decision:** Major revision

**Article Type:** original article.

**Corresponding Author:** Iván Dueñas-Espín

We would like to resubmit our original research article entitled "Development and validation of a scoring system to predict mortality in hospitalized patients with COVID-19: a retrospective cohort study in two large hospitals in Ecuador" for publication in PLOS ONE . We appreciate your patience throughout the review process, especially considering the delay caused by the transition to a new academic editor. We appreciate the valuable input and suggestions provided by the new editor, which have greatly improved the quality of our manuscript.

We also want to thank the previous reviewers for their thorough evaluation and constructive feedback. Your comments have been instrumental in strengthening our study. We are pleased to inform you that we have addressed all concerns and incorporated any necessary revisions into the manuscript.

We believe that the study's robust methodology, comprehensive analyses, and well-calibrated scoring system will be of great clinical utility in predicting mortality in hospitalized patients with COVID-19. We trust that the findings presented in this manuscript will provide valuable information to the scientific community and have important implications for patient care.

**COMMENTS TO THE AUTHOR**

**Editor’s comments**

**The article is very interesting, and I believe merits publication. However, there are some methodological issues that should be addressed before accepting it. I strongly suggest the authors review the TRIPOD statement recommendations for model development and validation and have a thorough look at the corresponding checklist.**

**ANSWER:**

Thank you for your input.

We have fulfil the TRIPOD statement as you can see in the new supplementary material “**TRIPOD Checklist: Prediction Model Development and Validation**” that allows the reader to check the accomplishment of all the statements for model validation.

We have added the **S1 Table. Tripod-Checklist** containing the TRIPOD statement checklist.

**The major aspect to include in the following version are the inclusion of measures of model calibration and accuracy (predictive values, likelihood ratios, overall accuracy) as well as the assessment of those measures in the comparison of the subpopulations in Guayaquil (sea-level population) and Quito (high altitude population).**

**ANSWER:**

Thank you for your insightful comments and suggestions. We understand the importance of evaluating not only the discrimination of our model but also its calibration. To address your concerns, we performed the calculations using Stata 16.1 with our Cox regression model, and included the abovementioned measures in our manuscript for showing model calibration and accuracy. In that regard, we have added next text in the Methods section (page 12, lines 247 to 258):

**Methods:**

**Calibration of the model in the validation cohort**

The calibration analysis of the model in the derivation dataset was performed using the Stata command "stcoxcal "[1]. These commands allowed us to assess the calibration of the Cox proportional hazards model by testing the intercepts and slopes against specified values. The "test" command tested the hypothesis that the intercepts are equal to 0 and the slopes are equal to 1, while the "trend" command tested the hypothesis that the slopes are equal to 1 with the intercepts estimated. These analyses were conducted to evaluate the goodness of fit and assess the agreement between the observed and predicted event probabilities, providing valuable insights into the model's calibration performance. Furthermore, we employed the "trend" command in Stata to assess the calibration of the Cox proportional hazards model in the validation dataset. Unlike the "test" command, the "trend" command allows for examining the overall trend of calibration over time.

This is the new text in Results section:

**Results (page 21, lines 375 to 381):**

The results of the calibration analysis in the validation dataset indicated that the model demonstrated good calibration overall (**Table 5**). The chi-square tests for intercepts and slopes did not provide evidence of miscalibration (Test 1: p=0.9321; Test 2: p=0.7206). Furthermore, the joint test of intercepts and slopes also showed no significant evidence of miscalibration (Test 3: p=0.9739). These findings suggest that the model's predicted event probabilities align well with the observed event probabilities in the validation dataset.

***Table 5. - Results of the calibration analysis in the validation dataset. The intercept test, slope test, joint test, and interaction test were performed to evaluate the model's calibration.***

| **Test** | **Chi-square** | **p-value** |
| --- | --- | --- |
| Intercept test | 0.14 | 0.9321 |
| Slope test | 0.13 | 0.7206 |
| Joint test | 0.22 | 0.9739 |
| Interaction test | 0.03 | 0.8729 |

**Discussion (page 26, lines 537 to 542):**

Moreover, the calibration analysis of the Cox proportional hazards model in the validation dataset demonstrated its ability to accurately estimate event probabilities. This indicates that the model can be considered reliable for predicting outcomes in similar population; Moreover, the tests of calibration, including the interaction between the slope and times, did not show significant differences, supporting the model's adequacy in terms of calibration over time.

Additionally, we recognize the need to include measures of model calibration and accuracy, as well as assessing those measures in the comparison of the subpopulations in Guayaquil (sea-level population) and Quito (high altitude population).In that regard, we have added next text in the methods section (page 13, lines 264 to 275):

In the calibration assessment of the Cox model applied to Quito, non-significant coefficients were found across all the tests, as evidenced by p-values greater than 0.05. Additionally, chi-square tests further reinforced these findings, with the null hypotheses not being rejected in all cases. These results suggest that the model is well-calibrated for this high-altitude city, as the model predictions do not significantly differ from the observed data. Therefore, the Cox model demonstrates an acceptable predictive accuracy for the city of Quito. Unfortunately, the application of the stcoxcal command for the Cox model to Guayaquil was hampered due to the limited number of observations in the original (non-imputed) dataset. This unfortunate circumstance restricts the evaluation of the calibration and predictive model for this city, which underscores the need to collect additional data to provide a more complete understanding of the model's applicability to Guayaquil.

**S4 Table. - Summary of the Cox Model Calibration Results for Quito**

| **Test** | **Variable** | **Coefficient** | **Standard Error** | **Z-score** | **P-value** | **95% Confidence Interval** |
| --- | --- | --- | --- | --- | --- | --- |
| Test 1 | _times (1) | -0.0891 | 0.5176 | -0.17 | 0.863 | -1.1035, 0.9254 |
| Test 1 | _times (2) | -0.0832 | 0.3581 | -0.23 | 0.816 | -0.7851, 0.6187 |
| Test 1 | Chi-square test |  |  |  | 0.9712 |  |
| Test 2 | _clogF | 0.9630 | 0.2842 | 3.39 | 0.001 | 0.4060, 1.5199 |
| Test 2 | _times (1) | -0.1389 | 0.7666 | -0.18 | 0.856 | -1.6413, 1.3635 |
| Test 2 | _times (2) | -0.1229 | 0.5541 | -0.22 | 0.824 | -1.2089, 0.9630 |
| Test 2 | Chi-square test |  |  |  | 0.8963 |  |
| Test 3 | Chi-square test |  |  |  | 0.9965 |  |
| Test 4 | 2._times | 0.1727 | 0.9667 | 0.18 | 0.858 | -1.7220, |
| Table notes:   1. Coefficients, standard errors, Z-scores, p-values, and 95% confidence intervals were obtained using the stcoxcal command in Stata. 2. All p-values are greater than 0.05, indicating a good calibration of the model for the city of Quito. 3. The null hypothesis of the Chi-square tests could not be rejected in all cases, further suggesting a well-calibrated model. 4. The variables starting with '_times' represent different time points, 'clogF' is the estimated cumulative hazard, and '_cons' refers to the constant term. '2._times#c._clogF' is the interaction term between time point 2 and the estimated cumulative hazard. | | | | | | |

**Reference:**

1. Royston P. Tools for checking calibration of a Cox model in external validation: Prediction of population-averaged survival curves based on risk groups. Stata J. 2015;15(1):275–91.

**Specific comments are shown below.**

**ABSTRACT**

**The best cutoff point, including at least sensitivity and specificity (and possibly predictive values and LR) should be included. Sample size and statistical analysis should also be included. The conclusion should be related to the accuracy of the prediction model itself beyond the variables included.**

We have added nexts text in the “Abstract” section (page 3, line 47 a 55):

“… a cutoff score ≥39 points demonstrates superior performance with a sensitivity of 93.10%, a specificity of 70.28%, and a correct classification rate of 72.66%. The LR+ (3.1328) and LR- (0.0981) values further support its efficacy in identifying high-risk patients.”

Regarding the sample we corrected as follows (page 14, line 290):

We analyzed 5,062 analyzed hospitalized patients with COVID-19 treated at two hospitals.

Regarding including statistical analysis, we added (p3, lines 35 to 36):

Statistical analyses were conducted on an imputed dataset, involving the development of a Cox proportional hazards regression model, assessment of goodness of fit, discrimination, and calibration.

We have added nexts text in the “Results” section (page 23, lines 385 to 392):

Furthermore, a cutoff score of ≥39 is considered a better cutoff over other scores because of its balance between sensitivity and specificity, along with other performance indicators. With a cut-off score of 39, a sensitivity of 93.10%, a specificity of 70.28% and a correct classification of 72.66% are achieved. In addition, the LR+ (3.1328) and LR- (0.0981) values support the usefulness of the cutoff score of 39 in identifying patients at high risk of mortality. These indicators suggest that a cut-off score of 39 provides a reasonable balance between detecting patients at risk and identifying those who are not, offering good discriminatory power.

You can see the results of each accuracy measure in this table:

**Summary of Calibration and Accuracy Measures of the Prediction Model (validation database)**

| **Cutoff Score** | **Sensitivity** | **Specificity** | **LR+** | **LR-** | **Overall Accuracy** |
| --- | --- | --- | --- | --- | --- |
| >= 0 | 100.00% | 0.00% | 1.0000 |  | 10.43% |
| >= 6 | 100.00% | 3.21% | 1.0332 | 0.0000 | 13.31% |
| >= 7 | 100.00% | 4.42% | 1.0462 | 0.0000 | 14.39% |
| >= 8 | 100.00% | 5.22% | 1.0551 | 0.0000 | 15.11% |
| >= 9 | 100.00% | 8.84% | 1.0969 | 0.0000 | 18.35% |
| >= 10 | 100.00% | 11.24% | 1.1267 | 0.0000 | 20.50% |
| >= 13 | 100.00% | 11.65% | 1.1318 | 0.0000 | 20.86% |
| >= 14 | 100.00% | 13.65% | 1.1581 | 0.0000 | 22.66% |
| >= 15 | 100.00% | 20.88% | 1.2640 | 0.0000 | 29.14% |
| >= 16 | 100.00% | 22.89% | 1.2969 | 0.0000 | 30.94% |
| >= 17 | 100.00% | 26.10% | 1.3533 | 0.0000 | 33.81% |
| >= 19 | 100.00% | 28.11% | 1.3911 | 0.0000 | 35.61% |
| >= 21 | 100.00% | 28.92% | 1.4068 | 0.0000 | 36.33% |
| >= 22 | 100.00% | 33.73% | 1.5091 | 0.0000 | 40.65% |
| >= 23 | 100.00% | 34.94% | 1.5370 | 0.0000 | 41.73% |
| >= 24 | 100.00% | 40.56% | 1.6824 | 0.0000 | 46.76% |
| >= 25 | 96.55% | 42.97% | 1.6931 | 0.0802 | 48.56% |
| >= 26 | 96.55% | 45.38% | 1.7677 | 0.0760 | 50.72% |
| >= 27 | 96.55% | 47.39% | 1.8352 | 0.0728 | 52.52% |
| >= 28 | 96.55% | 47.79% | 1.8493 | 0.0722 | 52.88% |
| >= 29 | 96.55% | 49.80% | 1.9233 | 0.0692 | 54.68% |
| >= 30 | 96.55% | 51.41% | 1.9869 | 0.0671 | 56.12% |
| >= 31 | 96.55% | 56.22% | 2.2056 | 0.0613 | 60.43% |
| >= 32 | 96.55% | 56.63% | 2.2261 | 0.0609 | 60.79% |
| >= 33 | 96.55% | 59.84% | 2.4041 | 0.0576 | 63.67% |
| >= 34 | 96.55% | 61.04% | 2.4785 | 0.0565 | 64.75% |
| >= 35 | 96.55% | 63.45% | 2.6419 | 0.0543 | 66.91% |
| >= 36 | 96.55% | 66.67% | 2.8966 | 0.0517 | 69.78% |
| >= 37 | 96.55% | 67.07% | 2.9319 | 0.0514 | 70.14% |
| >= 38 | 93.10% | 69.08% | 3.0107 | 0.0998 | 71.58% |
| >= 39 | 93.10% | 70.28% | 3.1328 | 0.0981 | 72.66% |
| >= 40 | 89.66% | 73.09% | 3.3320 | 0.1415 | 74.82% |
| >= 41 | 89.66% | 73.49% | 3.3824 | 0.1408 | 75.18% |
| >= 42 | 86.21% | 77.11% | 3.7659 | 0.1789 | 78.06% |
| >= 43 | 86.21% | 77.91% | 3.9028 | 0.1770 | 78.78% |
| >= 44 | 82.76% | 80.72% | 4.2931 | 0.2136 | 80.94% |
| >= 45 | 79.31% | 83.94% | 4.9371 | 0.2465 | 83.45% |
| >= 46 | 75.86% | 84.34% | 4.8435 | 0.2862 | 83.45% |
| >= 47 | 72.41% | 85.14% | 4.8733 | 0.3240 | 83.81% |
| >= 48 | 72.41% | 85.54% | 5.0086 | 0.3225 | 84.17% |
| >= 49 | 68.97% | 87.55% | 5.5395 | 0.3545 | 85.61% |
| >= 50 | 65.52% | 87.95% | 5.4379 | 0.3921 | 85.61% |
| >= 51 | 55.17% | 91.57% | 6.5419 | 0.4896 | 87.77% |
| >= 52 | 55.17% | 91.97% | 6.8690 | 0.4874 | 88.13% |
| >= 53 | 44.83% | 91.97% | 5.5810 | 0.5999 | 87.05% |
| >= 54 | 41.38% | 92.37% | 5.4229 | 0.6346 | 87.05% |
| >= 55 | 27.59% | 93.57% | 4.2931 | 0.7739 | 86.69% |
| >= 56 | 24.14% | 95.18% | 5.0086 | 0.7970 | 87.77% |
| >= 58 | 20.69% | 95.58% | 4.6834 | 0.8298 | 87.77% |
| >= 59 | 20.69% | 95.98% | 5.1517 | 0.8263 | 88.13% |
| >= 61 | 20.69% | 97.19% | 7.3596 | 0.8160 | 89.21% |
| >= 63 | 20.69% | 98.39% | 12.8793 | 0.8061 | 90.29% |
| >= 65 | 20.69% | 98.80% | 17.1724 | 0.8028 | 90.65% |
| >= 66 | 20.69% | 99.60% | 51.5176 | 0.7963 | 91.37% |
| >= 68 | 17.24% | 99.60% | 42.9313 | 0.8309 | 91.01% |
| >= 69 | 13.79% | 99.60% | 34.3451 | 0.8655 | 90.65% |
| >= 72 | 10.34% | 99.60% | 25.7588 | 0.9002 | 90.29% |
| >= 73 | 10.34% | 100.00% |  | 0.8966 | 90.65% |
| >= 77 | 3.45% | 100.00% |  | 0.9655 | 89.93% |

Now, regarding the the best cutoff point for Quito and Guayaquil, we have added next text in the Results section (page 24, lines 400 to 407):

Results:

When comparing between both hospitals, (S10 Table) there were differences in sensitivity, specificity, correct classification, LR+ (positive likelihood ratio) and LR- (negative likelihood ratio) between the populations of Guayaquil and Quito at each cut-off point of the COVD-19 in-hospital score tested (only show selected cut points for simplicity). It is observed that, in Guayaquil, the selected score points had higher sensitivity values, but lower specificity compared to those in Quito. In addition, the score in Guayaquil exhibited lower overall precision and more modest likelihood ratios than the score in Quito.

Discussion (page 25, lines 517 to 523):

Moreover, we strongly recommend the external validation of the “COVID-19 in-hospital mortality score” in independent populations to assess its generalizability and applicability across different settings. Future validation studies involving diverse patient cohorts will provide valuable insights into the performance and robustness of the scoring system, ensuring its reliability and usefulness in clinical practice.

**S12 Table. - Comparative detailed report of sensitivity and specificity between Guayaquil and Quito.**

| **Detailed report of sensitivity and specificity for Guayaquil** | | | | | |
| --- | --- | --- | --- | --- | --- |
| **Cutpoint** | **Sensitivity** | **Specificity** | **Correctly Classified** | **LR+** | **LR-** |
| ( >= 35 ) | 94.55% | 29.91% | 56.25% | 1.3489 | 0.1823 |
| ( >= 36 ) | 93.59% | 31.77% | 56.96% | 1.3717 | 0.2017 |
| ( >= 37 ) | 93.20% | 33.06% | 57.56% | 1.3922 | 0.2058 |
| ( >= 38 ) | 92.43% | 35.15% | 58.49% | 1.4253 | 0.2154 |
| ( >= 39 ) | 91.82% | 36.96% | 59.31% | 1.4564 | 0.2214 |
| ( >= 40 ) | 90.65% | 39.32% | 60.24% | 1.4940 | 0.2377 |
| ( >= 41 ) | 89.52% | 42.16% | 61.46% | 1.5477 | 0.2487 |
| ( >= 42 ) | 86.71% | 45.42% | 62.24% | 1.5887 | 0.2926 |
| ( >= 43 ) | 85.78% | 47.90% | 63.33% | 1.6464 | 0.2968 |
| **Detailed report of sensitivity and specificity for Quito** | | | | | |
| **Cutpoint** | **Sensitivity** | **Specificity** | **Correctly Classified** | **LR+** | **LR-** |
| ( >= 35 ) | 88.23% | 61.04% | 64.20% | 2.2644 | 0.1929 |
| ( >= 36 ) | 87.26% | 63.33% | 66.12% | 2.3798 | 0.2012 |
| ( >= 37 ) | 86.12% | 64.46% | 66.98% | 2.4231 | 0.2153 |
| ( >= 38 ) | 83.86% | 66.61% | 68.61% | 2.5110 | 0.2424 |
| ( >= 39 ) | 82.78% | 67.75% | 69.50% | 2.5667 | 0.2542 |
| ( >= 40 ) | 79.43% | 70.52% | 71.55% | 2.6940 | 0.2917 |
| ( >= 41 ) | 78.02% | 71.65% | 72.39% | 2.7519 | 0.3067 |
| ( >= 42 ) | 75.16% | 75.96% | 75.87% | 3.1270 | 0.3270 |
| ( >= 43 ) | 74.19% | 77.18% | 76.83% | 3.2510 | 0.3344 |
| Note: Sensitivity represents the proportion of true positive cases correctly identified. Specificity denotes the proportion of true negative cases correctly identified. LR+ indicates the strength of association between the score and mortality risk, while LR- signifies the strength of negative association. Accuracy refers to the overall correct classification rate based on the chosen cutoff point. | | | | | |

**METHODS**

**As the model is based on a cox regression , the sample size should be based on plausible hazard ratios and not in risk ratios.**

**ANSWER**

We appreciate your observation regarding the sample size calculation for our study, which is based on a Cox regression model. We understand that the sample size should be determined using plausible hazard ratios rather than risk ratios. We apologize for the oversight in our initial submission.

In the revised manuscript, we will update the sample size calculation to reflect the appropriate hazard ratios. We reevaluated the sample size requirements and provided a clear explanation of our calculations, ensuring that the assumptions are consistent with the Cox regression model.

As our developed score includes multiple predictor variables, it is challenging to provide a single realistic hazard ratio that represents the combined effect of all these variables. Instead, we could consider the hazard ratio for each variable separately and then interpret the results collectively.

With that said, here are some plausible hazard ratios based on similar studies or the clinical importance of each variable:

- Male sex: HR 1.2 - 1.5 (considering that male sex may have a moderately increased risk) (Zhou et al., 2020).
- Age categories: one might consider HRs of approximately 1.5 - 2.0 for each increase in age category, as age is a known risk factor for more severe outcomes (O'Driscoll et al., 2021).
- Hypoxia: HR 2.0 - 3.0 (hypoxia is a significant factor in the severity of COVID-19 and could have a substantial effect on risk) (Xie et al., 2020).
- Laboratory parameters: the HRs for these parameters may vary widely depending on the variable and the range of values considered. They may range from 1.1 - 2.0 for moderate changes in laboratory levels and potentially higher for extreme changes (Henry et al., 2020).

In that regard we choose a HR of 1.3, and, after revisiting the calculations using Stata 16.1, we have determined that our previous calculations using the GRANMO 7.12 program were not accurate. The new sample size calculation based on Cox PH regression with an alpha risk of 0.05, beta risk of 0.2, and a minimum hazard ratio of 1.3 yielded an estimated sample size of 3,258 with 457 expected events. We apologize for any inconvenience this may have caused and appreciate the opportunity to correct this error. We corrected the first paragraph of the statistical analyses (page 8, lines 172 to 175), as follows:

“For sample size calculation, based on Cox PH regression with an alpha risk of 0.05, beta risk of 0.2, and a minimum hazard ratio of 1.3 yielded an estimated sample size of 3,258 with 457 expected events. For the calculations, we used the program Stata 16.1 (StataCorp. Stata Statistical Software: Release 16. College Station, TX: StataCorp LLC; 2019).”

References:

Henry, B. M., de Oliveira, M. H. S., Benoit, S., Plebani, M., & Lippi, G. (2020). Hematologic, biochemical and immune biomarker abnormalities associated with severe illness and mortality in coronavirus disease 2019 (COVID-19): a meta-analysis. Clinical Chemistry and Laboratory Medicine (CCLM), 58(7), 1021-1028.

O'Driscoll, M., Ribeiro Dos Santos, G., Wang, L., Cummings, D. A. T., Azman, A. S., Paireau, J., ... & Salje, H. (2021). Age-specific mortality and immunity patterns of SARS-CoV-2. Nature, 590(7844), 140-145.

Xie, J., Covassin, N., Fan, Z., Singh, P., Gao, W., Li, G., ... & Somers, V. K. (2020). Association between hypoxemia and mortality in patients with COVID-19. Mayo Clinic Proceedings, 95(6), 1138-1147.

Zhou, F., Yu, T., Du, R., Fan, G., Liu, Y., Liu, Z., ... & Guan, L. (2020). Clinical course and risk factors for mortality of adult inpatients with COVID-19 in Wuhan, China: a retrospective cohort study. The Lancet, 395(10229), 1054-1062.

**Evaluation of a model should include measures of discrimination (as shown by the authors with ROC curves) , but also measures of CALLIBRATION, which are not included in the manuscript.**

**ANSWER:**

Thank you for your insightful comments and suggestions. We understand the importance of evaluating not only the discrimination of our model but also its calibration. Additionally, we recognize the need to include measures of model calibration and accuracy, as well as assessing those measures in the comparison of the subpopulations in Guayaquil (sea-level population) and Quito (high altitude population).

To address your concerns, we performed the calculations using Stata 16.1 with a Cox regression model, and included the abovementioned measures in our manuscript for showing model calibration and accuracy. So we added several parts in the manuscript:

we have added next text in the Methods section (page 12 and 13, lines 247 to 258):

Methods:

The calibration analysis of the model in the derivation dataset was performed using the Stata command "stcoxcal "[1]. These commands allowed us to assess the calibration of the Cox proportional hazards model by testing the intercepts and slopes against specified values. The "test" command tested the hypothesis that the intercepts are equal to 0 and the slopes are equal to 1, while the "trend" command tested the hypothesis that the slopes are equal to 1 with the intercepts estimated. These analyses were conducted to evaluate the goodness of fit and assess the agreement between the observed and predicted event probabilities, providing valuable insights into the model's calibration performance. Furthermore, we employed the "trend" command in Stata to assess the calibration of the Cox proportional hazards model in the validation dataset. Unlike the "test" command, the "trend" command allows for examining the overall trend of calibration over time.

Reference:

1. Royston P. Tools for checking calibration of a Cox model in external validation: Prediction of population-averaged survival curves based on risk groups. Stata J. 2015;15(1):275–91.

**How do the authors arrive to the best cutoff point (39 points as shown in result section)?There is no mention in the manuscript about that. In addition to sensitivity and specificity, other measures like overall accuracy, predictive values and likelihood ratios should be included at least for the best cutoff point.**

**ANSWER:**

Thank you very much, we have added next text in the abstract (page 3, lines 47 to 55):

moreover, a cutoff score ≥39 points demonstrates superior performance with a sensitivity of 93.10%, a specificity of 70.28%, and a correct classification rate of 72.66%. The LR+ (3.1328) and LR- (0.0981) values further support its efficacy in identifying high-risk .patients.

And we have added a text in Results section (page 21, lines 385 to 392) as follows:

Furthermore, a cutoff score of ≥39 is considered a better cutoff over other scores because of its balance between sensitivity and specificity, along with other performance indicators. With a cut-off score of 39, a sensitivity of 93.10%, a specificity of 70.28% and a correct classification of 72.66% are achieved. In addition, the LR+ (3.1328) and LR- (0.0981) values support the usefulness of the cutoff score of 39 in identifying patients at high risk of mortality. These indicators suggest that a cut-off score of 39 provides a reasonable balance between detecting patients at risk and identifying those who are not, offering good discriminatory power.

**Cut off points for defining risk categories (low, moderate, and high) cannot be based on score terciles. Instead, could be based on a predefined risk levels (for example less than 10% as low risk, 10-50% as intermediate risk and more than 50% as high risk) or likelihood ratios (positive likelihood ratio above 10 for high risk and negative likelihood ratio less than 0.1 as low risk).**

**ANSWER:**

Thank you for you input. We have reconsidered the cutoff points and recateorized the score in three categories as it is mentioned in the next paragaraph, that have been added to the methods section (page 11, lines 234 to 237):

Methods:

The categorization scheme for determining risk levels was established using the provided sensitivity, specificity, LR+, and LR- values. The derivation dataset was initially used to identify the cutoff points, and subsequently, the categorization scheme was applied to the independent validation dataset to evaluate its performance. This two-step approach ensures the reliability and generalizability of the risk classification system.

And another paragraph un the “Results” section (page 22, lines 408 to 416):

Results:

Also, and based on the sensitivity and specificity data provided, as well as the LR+ and LR- values, we can establish risk levels using the following cut-off points: low risk (score ≤ 30) with high specificity (56.18%) and LR- of 0.0000, indicating a high probability of negative results; moderate risk (score between 31 and 65) with a range of sensitivity (100% to 18.92%) and specificity (60.96% to 99.20%), suggesting a mix of positive and negative probabilities; and high risk (score > 65) with a sensitivity of 10.81%, a specificity of 99.20%, and an LR+ of 13.5675, indicating significantly higher odds of positive results compared with lower scores. These cut-off points help classify people into different levels of risk based on the balance between sensitivity and specificity.

Furthermore, we have changed the figures accordingly.

Regarding the sensitivity, specificity, overall accuracy, positive likelihood ratio and negative likelihood ratio, per each stratum of categorization with the score we have built the next supplementary table

**S11 Table. Sensitivity, specificity, and classification accuracy of the proposed risk classification cutpoints.**

| Cutpoint | Sensitivity | Specificity | Correctly Classified | LR+ | LR- |
| --- | --- | --- | --- | --- | --- |
| <= 30 | >96.55% | <51.41% | <56.12% | <1.9869 | >0.0671 |
| 31-65 | 96.55% to 20.69% | 56.22% to 98.80% | 60.43% to 90.65% | 2.2056 to 17.1724 | 0.0613 to 0.8028 |
| > 65 | <20.69% | > 98.80% | >90.65% | >17.1724 | <0.8028 |

**The authors stated: “We tested the cutoff value of the score in the validation cohort by calculating the AUC in ROC curve analyses”. However cutoff values are not tested using the AUC but looking at the overall accuracy, sensitivity, specificity, predictive values, and likelihood ratios of such proposed values.**

**ANSWER:**

Thank you. As we have mentioned above, we have checked the overall accuracy, sensitivity, specificity, predictive values, and likelihood ratios for each one of the proposed cutoffs in the categorization.

**Definition of hypoxemia (I would use the most accurate term hypoxemia instead of hypoxia) should be stated in method section according to the altitude (with a relevant reference)**

**ANSWER:**

Thank you, it has been done. We added nest text in the “Methods” section (pages 8 and 9, lines 160 to 169):

We also used the variable hypoxemia. Although there is no specific reference available for the altitude of Quito, efforts have been made to establish a consistent and justified criterion. The operational definition of hypoxemia was based on previous studies, such as the work of Luks and Swenson (2011) (21) and Hupperets et al. (2004) (22), who explored the effects of altitude on respiratory physiology and the hypoxic ventilatory response. In the absence of widely established literature defining Quito's altitude-specific hypoxemia, a consensus was reached based on clinical guidelines and expert opinion (23). According to this consensus, hypoxemia was defined as an oxygen saturation level below 92% in Quito. Additionally, for sea level (Guayaquil), a threshold below 95% was chosen to define hypoxemia (23).

References:

21. Luks AM, Swenson ER. Pulse oximetry at high altitude. High Alt Med Biol. 2011;12(2):109–19.

22. Hupperets MDW, Hopkins SR, Pronk MG, Tiemessen IJH, Garcia N, Wagner PD, et al. Increased hypoxic ventilatory response during 8 weeks at 3800 m altitude. Respir Physiol Neurobiol. 2004;142(2–3):145–52.

23. Llano M, Villamagua B, Garelli DZ, Freund P, Francisco V, Fernando AL, et al. Biomedical journal. Biomed J. 2016;1(1):1–9.

**RESULTS**

**It is not possible that in hospital mortality rate is 2.4 deaths per 10000 person days since mortality was more than 20% and there were around 50000 person days of follow up.**

**ANSWER**

We apologize for the error in our previous statement. The correct information has been changed in the Results section:

Rather:

“A total of 5,062 patients were analyzed. Accumulated mortality during the study was 22.6% (1,139 of 5,062 patients died in six months), which represents an in-hospital mortality rate of 2.4 deaths per 10,000 person-days (considering a total of person-days of follow-up of 48,263)."

The correct information is:

“A total of 5,062 patients were analyzed. Cumulative mortality during the study was 22.6% (1,139 of 5,062 patients died in six months), which represents an in-hospital mortality rate of 236 deaths per 10,000 person-days (considering a total of person-days of follow-up of 48.263)."

We apologize for any confusion caused by the above incorrect statement. We also changed the Results section in the line 290.

**The full prediction model should be shown (at least as supplementary material) .**

**ANSWER:**

Thanks for your suggestion. We appreciate your interest in the full prediction model. While we provide a summary of the model results and key variables in the manuscript, we understand the value of sharing the full prediction model for greater scrutiny and transparency. Therefore, we will make the full prediction model available as supplementary material, ensuring that readers have access to all relevant details and can fully evaluate the model.

We added a **S6 Table. - Cox regression modelling with multiple imputations was applied**. with a text, as follows:

Cox regression modelling with multiple imputations was applied. The model was defined as follows:

h(t|x) = h0(t) * exp(b1 * sex + b2 * age in quartiles + b3 * hypoxemia + b4 * hospital glycaemia (categorical) + b5 * RCP (binomial) + b6 * pH (categorical) + b7 * AST:ALT ratio (categorical) + b8 * leucocytosis (categorical))

where:

h(t|x) represents the instantaneous hazard rate at time t, conditioned on the predictor variables x.

h0(t) is the baseline hazard function at time t.

b1, b2, ..., b8 are the estimated coefficients for each of the predictor variables.

**Results should include evaluation of calibration. For the best cutoff point, results of overall accuracy, predictive values, LR + and -ve should be included. These analysis should also be shown for the subgroup analysis (Quito vs Guayaquil).**

**ANSWER**

As we mentioned above, and regarding the the best cutoff point for Quito and Guayaquil, we have added next text in the Results section (page 22, lines 400 to 407):

The cut point in Quito (UIO) and Guayaquil (GYE) were similar regarding the accuracy and other metrics.

**S10 Table. -** **Comparative detailed report of sensitivity and specificity between Guayaquil and Quito.**

| **Detailed report of sensitivity and specificity for Guayaquil** | | | | | |
| --- | --- | --- | --- | --- | --- |
| **Cutpoint** | **Sensitivity** | **Specificity** | **Correctly Classified** | **LR+** | **LR-** |
| ( >= 35 ) | 94.55% | 29.91% | 56.25% | 1.3489 | 0.1823 |
| ( >= 36 ) | 93.59% | 31.77% | 56.96% | 1.3717 | 0.2017 |
| ( >= 37 ) | 93.20% | 33.06% | 57.56% | 1.3922 | 0.2058 |
| ( >= 38 ) | 92.43% | 35.15% | 58.49% | 1.4253 | 0.2154 |
| ( >= 39 ) | 91.82% | 36.96% | 59.31% | 1.4564 | 0.2214 |
| ( >= 40 ) | 90.65% | 39.32% | 60.24% | 1.4940 | 0.2377 |
| ( >= 41 ) | 89.52% | 42.16% | 61.46% | 1.5477 | 0.2487 |
| ( >= 42 ) | 86.71% | 45.42% | 62.24% | 1.5887 | 0.2926 |
| ( >= 43 ) | 85.78% | 47.90% | 63.33% | 1.6464 | 0.2968 |
| **Detailed report of sensitivity and specificity for Quito** | | | | | |
| **Cutpoint** | **Sensitivity** | **Specificity** | **Correctly Classified** | **LR+** | **LR-** |
| ( >= 35 ) | 88.23% | 61.04% | 64.20% | 2.2644 | 0.1929 |
| ( >= 36 ) | 87.26% | 63.33% | 66.12% | 2.3798 | 0.2012 |
| ( >= 37 ) | 86.12% | 64.46% | 66.98% | 2.4231 | 0.2153 |
| ( >= 38 ) | 83.86% | 66.61% | 68.61% | 2.5110 | 0.2424 |
| ( >= 39 ) | 82.78% | 67.75% | 69.50% | 2.5667 | 0.2542 |
| ( >= 40 ) | 79.43% | 70.52% | 71.55% | 2.6940 | 0.2917 |
| ( >= 41 ) | 78.02% | 71.65% | 72.39% | 2.7519 | 0.3067 |
| ( >= 42 ) | 75.16% | 75.96% | 75.87% | 3.1270 | 0.3270 |
| ( >= 43 ) | 74.19% | 77.18% | 76.83% | 3.2510 | 0.3344 |

**Results of tests for proportional hazards and GOF should be mentioned.**

**ANSWER:**

Methods section (pages 10 and 11, lines 212 to 216):

The model was tested for both goodness of fit and the proportional hazards assumption. Goodness of fit was assessed using the Akaike Information Criterion (AIC) and the Bayesian Information Criterion (BIC) through the “estat ic” command. The proportional hazards assumption was tested with the “estat phtest” command, which involved running chi-square tests for each covariate included in the model.

Results section (page 17, lines 335 to 341)

Goodness of fit was assessed using the Akaike Information Criterion (AIC) and the Bayesian Information Criterion (BIC), which yielded AIC = 286.4317 and BIC = 330.3455. Chi-square tests for each variable, together with their corresponding p-values, suggest that the risks are proportional over time. The overall test p-value for the model was 0.9814, further supporting the proportional hazards assumption. These results demonstrate that the Cox regression model used in the analysis provides a good fit to the data and satisfies the proportional hazards assumption.

**“DISCUSSION
The most important predictor of hospitalization and mortality is vaccination. This score was developed on a population that was essentially not vaccinated. Therefore the usefulness of the score may be limited to those unvaccinated. The validation of the score in current settings with highly vaccinated population could be discussed.
The results are highly influenced by the fact of being hospitalized in high altitude setting. The score appears to work better in high altitude population. This has important implications, since most scoring systems have been developed in sea-level populations. In fact it appears that this particular score is not very useful for sea level population (however this issue should be assessed by evaluating sensitivity , specificity, and other accuracy measures is this subgroup).”**

**ANSWER**

Thank you for your valuable comments and suggestions on our manuscript titled "COVID-19 In-Hospital Mortality Score: A Predictive Tool for Unvaccinated and High-Altitude Populations." We appreciate the opportunity to address your concerns and have carefully considered your feedback.

The importance of vaccination as a key predictor of hospitalization and mortality is acknowledged, as well as the relevance of validating our score in the current context with a highly vaccinated population.

Firstly, it should be clarified that our study was conducted prior to the development and implementation of the vaccines for the disease in question. Consequently, the study population consisted of unvaccinated individuals. However, it is understood that the utility of our score may be limited in the vaccinated population, and it would be valuable to investigate its applicability in that context. This observation is appreciated, and it is proposed to include a discussion in the limitations section of the article, highlighting the need for future research to assess the validity and utility of our score in vaccinated populations.

Secondly, it is agreed that our score appears to have greater utility in populations residing in high-altitude areas. This finding is relevant, as most scoring systems have been developed in sea-level populations. It is proposed to include a discussion in our article that emphasizes the importance of this finding and its practical implications. Additionally, in response to your suggestion, sensitivity, specificity, and other accuracy measures will be evaluated in the sea-level subpopulation to determine the utility of our score in this group, and these results will be added to the corresponding section of the article.

As you rightly pointed out, the most significant predictor of hospitalization and mortality is vaccination. We acknowledge that our score was developed primarily based on a population that was essentially unvaccinated. Consequently, the usefulness of the score may be limited to unvaccinated individuals. We have revised our manuscript to emphasize this limitation and have included a discussion on the importance of validating the score in settings with highly vaccinated populations.

Regarding the influence of high altitude on the results, we concur that the score appears to work better in high-altitude populations. We have highlighted this finding in our manuscript, noting the implications and the novelty of a scoring system developed for high-altitude populations, as most existing systems have been developed in sea-level populations. We also acknowledge the potential limitations of our score in sea-level populations and have added a section in the discussion to address the need for evaluating the sensitivity, specificity, and other accuracy measures in this subgroup.

We hope that these revisions address your concerns and strengthen our manuscript. We look forward to your feedback and the opportunity to further improve our work.

We have added two paragraphs in the discussion section (page 23 and 24, lines 441 to 482) as follows:

In the presented study, it was observed that the "COVID-19 in-hospital mortality score" demonstrated greater utility in predicting mortality in unvaccinated patients and those residing in higher altitude areas. It is important to note that this finding may be related to specific biological adaptations and environmental factors in these populations [53,54]. However, further research in diverse populations and contexts is required to confirm and better understand these findings.

Higher altitude populations have been shown to exhibit a lower rate of COVID-19 related mortality in some studies (31). This could be due to biological adaptations to hypoxia, which may play a role in the body's response to the virus. Additionally, specific environmental factors of high-altitude areas could influence the spread and severity of the disease (32). On the other hand, in unvaccinated populations, the lack of immunity acquired from vaccination might lead to more severe disease outcomes, which could explain the higher utility of the score in these populations.

Nevertheless, it was found that the score exhibited lower discriminative ability when analyzed in patients from Guayaquil's hospital and in women [S2 Table]. This suggests that the score might be less effective in certain population groups and contexts, emphasizing the importance of validating and adapting the score to different populations and settings.

In conclusion, although the "COVID-19 in-hospital mortality score" appears to be particularly useful in unvaccinated patients and those residing in higher altitude areas, further research is necessary to validate and adapt this score to different populations and contexts. This would enhance its applicability and utility in clinical practice and healthcare resource planning.

References:

(31) Arias-Reyes, C., Zubieta-DeUrioste, N., Poma-Machicao, L., Aliaga-Raduan, F., Carvajal-Rodriguez, F., Dutschmann, M., ... & Soliz, J. (2020). Does the pathogenesis of SARS-CoV-2 virus decrease at high-altitude?. Respiratory Physiology & Neurobiology, 103443.

(32) Simbaña-Rivera K, Jaramillo PRM, Silva JVV, Gómez-Barreno L, Campoverde ABV, Novillo Cevallos JF, et al. High-altitude is associated with better short-term survival in critically ill COVID-19 patients admitted to the ICU. PLoS One. 2022;17(3):e0262423.

**CONCLUSION**

**The objective was to develop a predictive model for mortality in patients hospitalized due to COVID-19. In that sense, the conclusion should answer the objective addressing the usefulness and limitations of the model developed.**

**ANSWER:**

Thanks for your comment. We recognize the importance of addressing the objective of our study in the conclusion. We will ensure that the conclusion explicitly discusses the utility and limitations of the predictive model for mortality in hospitalized patients with COVID-19.

We have added this text to the abstract (page 4, lines 58 to 61):

A statistically significant Cox regression model with strong discriminatory power and good calibration was developed to predict mortality in hospitalized patients with COVID-19, highlighting its potential clinical utility.

… and conclusion (page 28, lines 586 to 595):

Based on the statistical significance of the Cox regression model, its discriminatory ability (AUC-ROC), and calibration, the results suggest that the developed model is a valuable tool for predicting mortality in hospitalized COVID-19 patients. The model demonstrates robust statistical performance, with significant associations between the predictor variables and mortality outcomes. Furthermore, the model exhibits good discrimination, as evidenced by a high AUC-ROC value. The calibration analysis confirms the model's ability to accurately estimate mortality probabilities. These findings collectively indicate the potential clinical utility of the model in identifying individuals at higher risk of mortality and informing appropriate interventions.

**OTHER COMMENTS FROM REVIEWER 3**

**Reviewer #3: The cited website link for Ref #1 is broken and number of deaths due to are inaccurate (https://covid19.who.int). Please fix it.**

**ANSWER:**

Thank you. We have corrected:

World Health Organization (WHO) has reported a global total of 767,984,989 confirmed cases of COVID-19 caused by Severe acute respiratory syndrome coronavirus 2 (SARS-CoV-2), with 6,943,390 deaths. Additionally, as of 13 June 2023, a total of 13,397,292,784 vaccine doses have been administered worldwide (1).

Reference:

(1) WHO. WHO Coronavirus (COVID-19) Dashboard With Vaccination [Internet]. World Health Organization. 2021. Available from: https://covid19.who.int/
